# Mycobiota associated with strawberry fruits, their mycotoxin potential and pectinase activity

**DOI:** 10.1080/21501203.2020.1759719

**Published:** 2020-05-13

**Authors:** Mohamed A. Hussein, Ahmed H.M El-Said, Asmaa S. Yassein

**Affiliations:** Botany and Microbiology Department, Faculty of Science, South Valley University, Qena, Egypt

**Keywords:** Strawberry, aflatoxin, ochratoxin, pectinase activity

## Abstract

Forty-three species and variety belonging to 15 genera were collected from 30 strawberry fruit samples on Glucose-Czapek’s agar medium. Among them, *Aspergillus flavus, Aspergillus niger* and *Penicillium citrinum* were the most frequent species recovered from 53.3%, 70.0% and 50.0% of the samples, respectively. According to the ITS rDNA sequence, we confirmed the morphological identification result. Moreover, aflatoxin biosynthesis gene *omt-A* was detected in *A. flavus*, while *Aopks* gene was found in *A. niger*. Interestingly, we could not detect any aflatoxin or ochratoxin biosynthesis genes in the *P. citrinum* strain. The concentration of detected aflatoxin was 3.5 ppb produced by *A. flavus*, while *A. niger* gave 4.1 ppb as ochratoxin. *A. flavus* was the most pectinase producer among the selected strains, and the highest amount was obtained at 30°C after 6 days of incubation with initial medium pH 8.

## Introduction

Strawberries (*Fragaria* × *ananassa*), belonging to the Rosaceae family, are considered to be most important fruits worldwide. They contain a big number of phenolic compounds which have antioxidant properties (Hipol et al. 2014). One of the limiting factors that influence its economic value is the relatively short shelf life caused by postharvest spoilage (Al-Najada and Gherbawy 2015). Fungi were considered the main reason for strawberry postharvest spoilage (Lopes et al. 2014). The fungal species causing postharvest spoilage in strawberries vary according to the region, weather and crop management (Maas 1998). In Egypt, the main fungal species that cause postharvest rot in strawberry are *Botrytis cinerea* and *Rhizopus stolonifer* (El-Mougy et al. 2008). Other fungi associated with rotting of strawberry fruit at postharvest stage are *Alternaria alternata, Aspergillus niger, Cladosporium, Mucor* spp., *Penicillium* spp., *Phytophthora cactorum, Sclerotinia sclerotiorum* and *Trichoderma* spp. (Saber et al. 2003; Embaby and Abd-Ellatif 2013; Jensen et al. 2013; Juan et al. 2016).

During food colonisation by fungi, some species may generate different mycotoxins that are carcinogenic, immunosuppressive, genotoxic, nephrotoxic and teratogenic (Petzinger and Weidenbach 2002). *Aspergillus flavus* and *A. parasiticus* are the main aflatoxin producers, while ochratoxins are produced by *Aspergillus ochraceus* group and *A*. section *Nigri* (Roehuck and Maxuitenko 1994; Varga et al. 1996; Heenan et al. 1998). The natural incidence of *A. flavus* on different crops and quantification of aflatoxins in fruits and fungal cultures were documented in several studies (Singh and Sumbali 2000; Sarkar et al. 2011; Kachapulula et al. 2019).

Several *Penicillium* species were documented as afla and ochratoxin producers (Patino et al. 2005; Cabanes et al. 2010). Molecular tools have been used to investigate the afla and ochratoxigenicity of *A. flavus* and *A. niger* fungi in different food and feed sources (Mayer et al. 2003; Somashekar et al. 2004; Abdel-Hadi et al. 2010; Agriopoulou et al. 2016). In this matter, different specific primers have been designed for several genes involved in the aflatoxin biosynthetic pathways, i.e., *aflR, nor, ord, omt* and *ver* (Scherm et al. 2005; Rodrigues et al. 2009; Ahlberg et al. 2015). The presence of four genes: *aflR, nor A, omt A* and *ver 1* in *A. flavus* toxigenic isolates were evaluated using quadruplex polymerase (Gherbawy et al. 2012; Bandyopadhyay et al. 2016). *Anpks* and *Aopks* genes were used for ochratoxin biosynthesis, the detection of these genes was investigated in different *A. niger* strains (Castella and Cabanes 2011; Reddy et al. 2013; El-Hamaky et al. 2016).

The wall of fruit cell is composed of high per cent of polysaccharides, approximately 90%, divided into cellulose, hemicellulose and three groups of pectin (McNeil et al. 1984; Nathalie 2006). The principal of fungal spoilage depends on secreting of extracellular lytic enzymes such as pectinases that degrade these polymers to release water and the plant’s other intracellular components for use as nutrients for their growth and finally lead to developing inedible, unwanted quality and fruit rotting (Miedes and Lorences 2004; Al-Hindi et al. 2011).

This work is aimed to identify the mycobiota associated with strawberry, detect afla and ochratoxin toxin-producing genes in certain species and evaluate their pectinase activity.

## Materials and methods

### Strawberry fruit samples

Thirty samples of strawberry fruits were collected from different markets in Qena city, Egypt. Samples were put separately in a sterile polyethylene bag sealed and kept in other bags which were also sealed. Samples were transported to a mycological laboratory for fungal analysis.

### Determination of mycobiota

Dilution plate method used for isolation of fungi. Known weight of the fruit was placed in autoclaved conical flasks containing 100 ml sterile distilled water. From the appropriate dilution, 1 ml was transferred to each sterile plate followed by addition of about 20 ml liquefied Glucose-Czapek’s agar medium (Smith and Dawson 1944; Al-Dory 1980). The plates were incubated at 28 ± 2°C for 5–7 days and the developing colonies were identified and counted. The numbers of colonies were calculated/g of fruits fresh weight.

### Molecular characterisation of selected isolates

Based on isolation results, three isolates were chosen randomly from the most common species (*A. flavus, A. niger* and *Penicillium citrinum*) for molecular identification. ITS1 and ITS4 primers were used for ITS region amplification from selected isolates as mentioned by White et al. (1990). The purified bands were sequenced using the sequencer Gene analyser 3121. The deduced sequence was edited and aligned. The sequencing data were compared with the Gene Bank database (http://www.ncbi.nlm.nih.gov/BLAST/), where a nucleotide blast program was chosen to identify the homology between the PCR fragments and the sequences on the GeneBank database. A phylogenetic tree was constructed by MEGA6 (Tamura et al. 2013) using the Neighbor joining method with 1000 bootstrap repeats.

### Detection of aflatoxin and ochratoxin producing genes

Four documented primers (*omt-A* and *Aopks*) were used for detection of afla and ochratoxin, producing genes. The sequences of primers were as following: *omt-AF* “5-GACCAATACGCCCACACAG-”3, *omt-AR* “5-CTTTGGTAGCTGTTTCTCGC-”3 (Shweta et al. 2013) and *Aopks-F* “5-CAGACCATCGACACTGCATGC-”3, *Aopks-R* “5-CTGGCGTTCCAGTACCATGAG-”3 (Reddy et al. 2013). Twenty-five μl as volume was used for PCR reaction by mixing 0.5 μl of DNA with 0.5 μM of each primer, 2.5 μl buffer, 1.5 μl MgCl_2,_ 0.5 μl dNTPs, 0.5 μl Taq polymerase (Jena Bioscience, Germany) and water was added to make the volume up to 25 μl. The reactions were done in a C1000TM Thermo Cycler BioRad, Germany, with initial denaturising at 94°C for 5 min, followed by 35 cycles of 1 min at 94°C, 1 min as annealing temperature at 59°C and 1 min at 72°C for *omt-A* (Deabes et al. 2018) and 30 cycles of 1 min at 94°C, 1 min at 58°C and 1 min at 72°C for *Aopks* (El-Hamaky et al. 2016). Ten minutes at 72°C was used as the final extension for two reactions. PCR products were checked on a 1.4% agarose gel, stained with ethidium bromide.

### Quantitative assessment of afla and ochratoxins

The selected isolates (*A. flavus, A. niger* and *P. citrinum*) were inoculated into flasks containing yeast extract sucrose medium (YES) containing: sucrose, 40 g; yeast extract 20 g and distilled water, 1000 ml. The cultivation was made erlenmeyer flasks containing 100 ml of the medium. After autoclaving, the flasks were inoculated with a single 6 mm disc cut out from the margin of a 5-day colony of the fungus grown on Glucose-Czapek’s agar medium. Flasks were incubated at 28 ± 2°C for 14 days (Gabal et al. 1994). The mycotoxin levels were estimated by the fluorometric method using aflatoxin or ochratoxin standards to fluorometer calibration before reading the content of sample toxins (Hansen 1993).

### Pectinolytic activity of selected fungi

Three isolates were screened for their abilities to produce extracellular pectinase as described by Saleem et al. (2012). Using a sterile cork borer (6 mm diameter) discs from pre-cultured strains were cut to inoculate sterile conical flask containing 50 ml liquid medium of Hankin et al. (1971). After incubation for 7 days at 28°C, the cultures were filtered, and the filtrates were used to determine pectinase activity (Ammar et al. 1995). The average diameter of clear zones (in mm) for selected strains was measured.

### Factors affecting pectinase production

*A. flavus* was found to be the most active pectinase producer. So, they employed to evaluate the effect of different ecological factors on pectinase production.

### Effect of temperature and time course

Flasks inoculated with *A. flavus* were incubated at different temperature degrees (20°C, 30°C and 40°C) for 14 days and the cultures were harvested at 48-hour intervals. Cultures were filtered then centrifuged at 5,000 rpm for 7 min. The clear supernatants were assayed for pectinase activity.

### Effect of pH values

Medium flasks were pre-adjusted to different pH levels ranging from 2 to 12 (0.1 N HCl or 0.1 NaOH) before inoculation with *A. flavus*. The inoculated flasks were incubated at 30°C for 8 days. Three flasks for each pH value were prepared. At the end of the incubation period, cultures were filtered, centrifuged at 4°C for 7 minutes at 15,000 rpm and the clear supernatants were assayed for pectinase activity according to Sherwood (1966).

### Statistical analysis

All experiments were carried out in triplicates. Data obtained were expressed as mean ± standard error of the mean (SEM).

## Results

### Mycobiota from Strawberry fruits

Forty-three species and variety belonging to 15 genera were collected from 30 strawberry fruit samples (Table 1). *Aspergillus,* represented by 7 species and variety, was the most frequent genus isolated from 96.6% of the samples comprising 52.9% of the total fungi. *A. flavus* and *A. niger* were the most prevalent species among *Aspergillus*. They were recovered from 53.3% to 70% of the samples matching 26.6% and 21.6% of the total fungi, respectively (Table 1).

**Table 1.** Average total counts (ATC, calculated per g fresh fruit), percentage counts (%C, calculated per total fungi), number of cases of isolation (NCI, out of 30 samples) and percentage frequency (%F, calculated per 30 samples) of various fungal genera and species recovered from strawberry fruits on glucose-Czapek’s agar at 28°C.

Genera and species	ATC	%C	NCI	%F
*Acremonium*	140	1.6	3	10
*A. cerealis*	20	0.2	1	3.3
*A. rutilum*	100	1.6	1	3.3
*A. strictum*	20	0.2	1	3.3
*Alternaria*	550	6.4	14	46.6
*A. alternata*	530	6.1	14	46.6
*A. brassicicola*	10	0.1	1	3.3
*A. citri*	10	0.1	1	3.3
*Aspergillus*	4580	52.9	29	96.6
*A. flavus* Link	2300	26.6	16	53.3
*A. fumigatus*	150	1.7	4	13.3
*A. niger*	1870	21.6	21	70
*A. ochraceus*	120	1.4	5	16.6
*A. sydowii*	60	0.7	3	10
*A. terreus*	40	0.5	4	13.3
*A. terrus* var. *aureus*	20	0.2	2	6.6
*A. versicolor*	20	0.2	2	2.6
*Cladosporium*	710	8.2	14	46.6
*C. cladosporioides*	700	8.1	14	46.6
*C. sphaerospermum*	10	0.1	1	3.3
*Emericella nidulans*	200	2.3	8	26.6
*Epicoccum purpurascens*	10	0.1	1	3.3
*Mucor hiemalis*	10	0.1	1	3.3
*Mycosphaerella tassiana*	20	0.2	1	6.6
*Myrothecium rordium*	10	0.1	2	3.3
*Penicillium*	2290	26.4	23	76.6
*P. aurantiogriseum*	160	1.8	5	16.6
*P. camembretii*	10	0.1	1	3.3
*P. citrinum*	780	9.0	15	50
*P. corylophilum*	10	0.1	1	3.3
*P. dendriticum*	400	4.6	3	10
*P. duclauxii*	20	0.2	1	3.3
*P. echinulatum*	130	1.5	4	13.3
*P. funiculosum*	370	4.3	5	16.6
*P. implicatum*	20	0.2	1	3.3
*P. oxalicum*	20	0.2	1	3.3
*P. pinophilum*	40	0.5	1	3.3
*P. purpurogenum*	110	1.3	6	20
*P. spinulosum*	70	0.8	4	13.3
*P. variabile*	20	0.2	2	6.6
*P. verruculosum*	10	0.1	1	3.3
*P. vinaceum*	10	0.1	1	3.3
*P. waksmanii*	50	0.6	3	10
*Phoma glomerata*	10	0.1	1	3.3
*Pleospora herbarum*	20	0.2	1	3.3
*Scytalidium* state of *hendersonula toruloidea*	10	0.1	1	3.3
Sterile mycelia	50	0.6	2	6.6
*Trichoderma koningii*	20	0.2	2	6.6
*Ulocladium*	30	0.3	3	10
*U. botrytis*	20	0.2	2	6.6
*U. atrum*	10	0.1	1	3.3
**Average total count**	8660			
**Number of genera**	15			
**Number of species**	43 + 1 var.			

After *Aspergillus, Penicillium* (17 species) occupied the second place, isolated from 76.6% of the samples, comprising 26.4% of the total fungi. The most prevalent species was *P. citrinum* recovered from 50% of the samples with 9% from the total fungal counts. *Alternaria* and *Cladosporium* shared the third place with the same frequencies (46.6% from the samples). The other remaining fungal genera and species were isolated in low frequencies and amounts (Table 1).

### Molecular identification of selected strains and phylogenetic analysis

**Figure 1.**
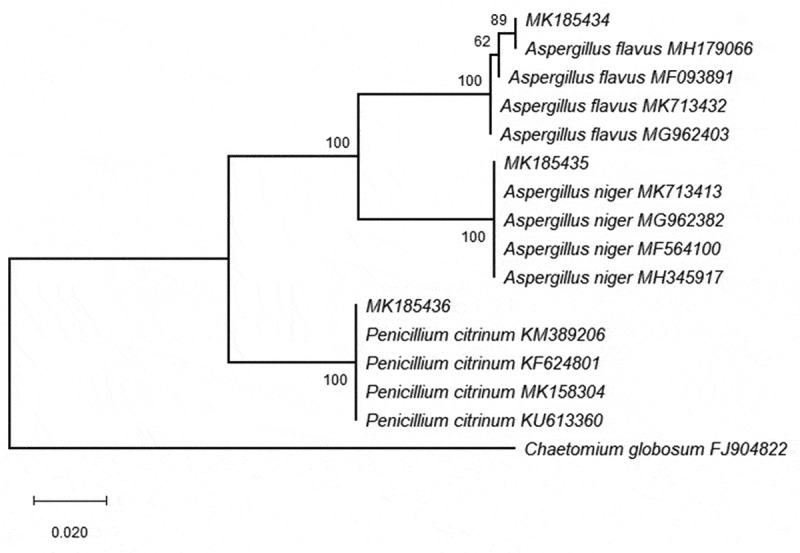
Phylogenetic relationship among three strains belonging to genus *Aspergillus* and *Penicillium* compared with some strains at NCBI.

The universal primers ITS 1 and ITS 4 were used to amplify the ITS regions of rDNA from selected isolates (*A. flavus, A. niger* and *P. citrinum*). The obtained sequences deposited in GenBank with accession numbers MK185434, MK185435 and MK185436 (Table 2). The phylogenetic analysis of tested strains with different sequences from National Centre for Biotechnology Information (NCBI) confirmed the morphological identification for the tested strains as *A. flavus, A. niger* and *P. citrinum* (Figure 1).

**Table 2.** Accession numbers, mycotoxin potential of selected fungal strains (in ppm) and their pectinolytic activity (in mm).

Fungal species	Accession No.	Aflatoxin	Ochratoxin	Pectinase activity
*Aspergillus flavus*	MK185434	3.5	ND	34
*A. niger*	MK185435	ND	4.1	28
*Penicillium citrinum*	MK185436	ND	ND	26

ND = not detected.

### Detection of aflatoxin and ochratoxin producing genes

**Figure 2.**
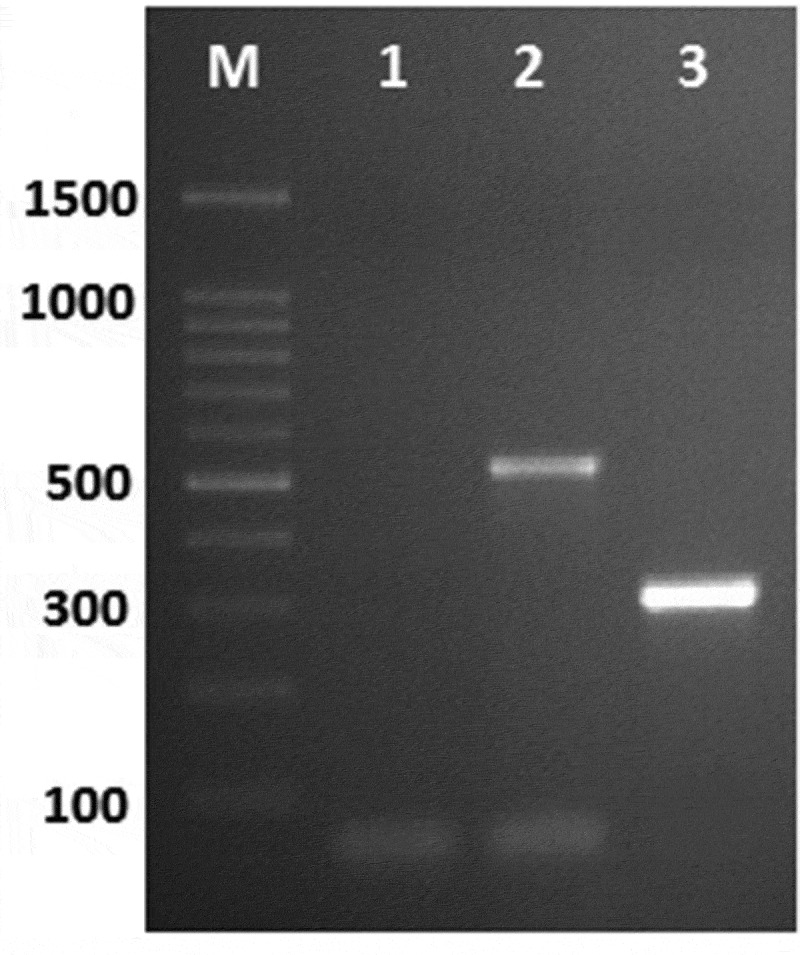
PCR amplification of *omt-A* and *Aopks* genes (300 and 549 bp) for tested isolates, whereas, Lane 1 = *P. citrinum*, 2 = *A. niger* and 3 *= A. flavus.*

Polymerase chain reaction (PCR) was applied using four sets of primers for the detection of different genes involved in aflatoxin and ochratoxin biosynthetic pathway. Bands of the fragments of *omt-A* and *Aopks* genes can be visualised at 300 and 549 bp, respectively (Figure 2). Aflatoxigenic *A. flavus* strain showed 300 bp DNA fragments that corresponded to the complete of aflatoxin B1 producing gene. In addition, *A. niger* isolate showed the complete of investigated ochratoxin genes. The obtained data exhibited complete absence of afla or ochratoxin genes in *P. citrinum*.

### Quantitative assessment of afla and ochratoxins

Data in Table 2 illustrate *A. flavus* was aflatoxigenic producer, while *A. niger* exhibited ochratoxigenic activity, but *P. citrinum* failed to be afla or ochratoxigenic producer. The data obtained from fluorometer showed the amount of aflatoxin produced by *A. flavu*s was 3.5 ppb, while the amount of ochratoxin by *A. niger* was 4.1 ppb.

### Pectinolytic activity

**Figure 3.**
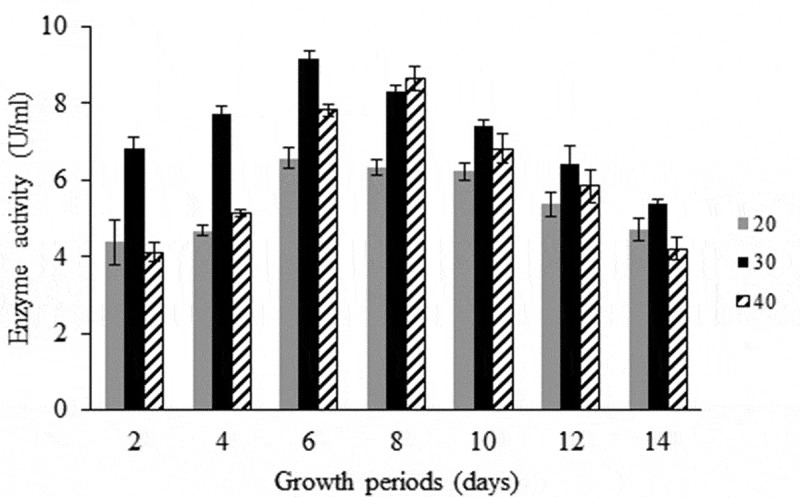
Effect of time course and temperatures on pectinase enzyme production by *A. flavus.*

**Figure 4.**
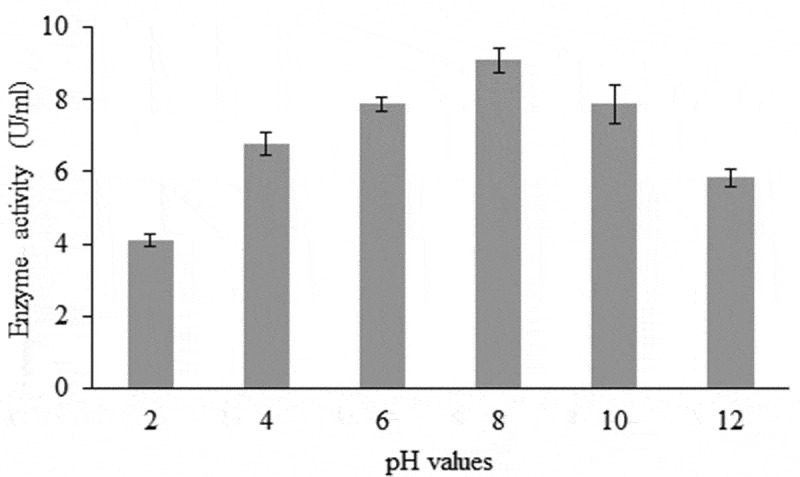
Effect of different pH values on pectinase enzyme production by *A. flavus.*

The three fungal species were screened for their abilities to produce pectinase enzyme using the cup-plate method. All tested isolates were pectinase producers, but with variable amounts (Table 2). In the first place, come *A. flavus* (34 mm) as the most active pectinase producer followed by *A. niger* (28 mm) and *P. citrinum* (26 mm). The activity of pectinase has been found to be affected by variable environmental conditions. The amount of pectinase produced by *A. flavus* was increased by extending the incubation period at different temperatures showing high levels at 6 days (20°C and 30°C) and 8 days (40°C). In general, the maximum production of the enzyme by *A. flavus* was achieved after 6 days at 30°C as the incubating temperature (Figure 3). The highest level of pectinase could be synthesised when the culture medium of the fungal species initially adjusted to pH 8. Considerable amounts of the enzyme were also estimated at pH 6 and pH 10, while low amounts of the enzyme were observed in cultures with more alkalinity or acidity (Figure 4).

## Discussion

Strawberry is the most important fruit containing high amount of vitamin C (Embaby et al. 2016). Their decay is caused by several fungi. Our result indicated that *A. flavus, A. niger* and *P. citrinum* were the most frequent species that contaminated strawberry fruits. This result conflicts with many studies which indicated that *Botrytis cinerea* is the main causative agent for strawberry fruit decay (Embaby et al. 2016; Mouden et al. 2016). Abdelfattah et al. (2016) reported that *Botrytis* and *Cladosporium* were the most common fungi from mature and immature strawberry fruits. On the other hand, *A. niger, B. cinerea, Penicillium digitatum* and *Rhizopus stolonifera* were considered as postharvest pathogenic fungi which could reduce the shelf life of strawberry fruit (Nabigol and Morshedi 2011). *A. alternata, A. niger, Penicillium* spp. and other fungal species were previously isolated from strawberry (Saber et al. 2003). Based on ITS region, the conventional identification was confirmed by molecular technique. The phylogenetic analysis revealed that our isolates were recognised to be *A. flavus, A. niger* and *P. citrinum*. The molecular characterisation of different fungi to species level using ITS was used by many investigators (Gherbawy et al. 2016; Abd Elhamid et al. 2017; Alsohaili and Bani-Hasan 2018).

The appearance of 300 and 549 bp bands with *A. flavus* and *A. niger* revealed the presence of aflatoxin B_1_ producing gene in *A. flavu*s and ochratoxin gene in *A. niger;* on the contrary, these genes are absent in *P. citrinum*. The presence of two targeted genes confirmed the abilities of tested isolates to produce aflatoxins and ochratoxin as previously mentioned by other researchers (Reddy et al. 2013; El-Hamaky et al. 2016; Sireesha et al. 2017). Ochratoxin producing genes were detected in *A. ochraceus, A. alliaceus, A. sclerotiorum, A. sulphureus, A. albertensis, A. auricomus*, and *A. wentii* strains. Gherbawy et al. (2012) documented the presence of aflatoxin and ochratoxin coding genes in 38.8% and 25% of *A. flavus* and *A. niger* isolates recovered from date palm, respectively. The concentration of aflatoxins in tested *A. flavus* isolates recovered from strawberry was ranged between 25.8 and 75.2 ng/ml (Saleem 2017). On the other hand, Gherbawy and Shebany (2018) found that 45.5% of *P. citrinum* isolates were ochratoxin A producers with amounts ranging from 1.3 to 2.5 μg/L.

*A. flavus* was the most active pectinase producer. These results fully agree with those obtained by Reddy and Sreeramulu (2012), who found that among four different fungi screened for pectinolytic activity, *A. flavus* was the most active producer of pectinase. Among *Aspergillus fumigatus, A. flavus, A. niger, A. ochraceus, A. oryzae, A. sydowii, Trichothecium* sp., *Penicillium* sp., *Trichoderma harzianum* and *T. viride, A. flavus* was able to produce pectinolytic enzyme (Usha et al. 2014). The higher amount of pectinase production by *A. flavus* was after 6 days of incubation at 30°C with pH 8 for the medium. In this respect, Yadav et al. (2007) reported that the highest yield of pectinase by *A. flavus* and *A. phoenicis* was obtained after 6 days, while in *A. niger* and *A. wentii* it was obtained after 8 days of incubation. Also, Palaniyappan et al. (2009) found the optimum temperature for pectinase production by *A. niger* MTCC 281 fungi was 30°C. Identical results were obtained by Yadav et al. (2007, 2008) who found that pH 8 is optimum for pectinase production in culture filtrate of *A. flavus*. Also, the optimum pH for pectinase activity by *A. niger* was 8.0 (Batool et al. 2013).

In conclusion, *A. flavus, A. niger* and *P. citrinum* constituted 26.6%, 21.6% and 9.0% of the total fungi recovered from strawberry fruits in Qena city, respectively. The tested strains of *A. flavus* and *A. niger* were afla and ochratoxin producers, respectively, but *P. citrinum* could not produce afla or ochratoxin. In addition, *A. flavus* was the most active pectinase producer and the maximum amount was achieved after 6 days of incubation at 30°C and pH of medium equal pH 8.
